# What are the functionalities and features of mobile health record apps supporting persons experiencing social exclusion? A systematic literature review

**DOI:** 10.3389/fdgth.2025.1629289

**Published:** 2025-09-15

**Authors:** Felicien Izaturwanaho, Marie E. Ward, Clíona Ní Cheallaigh, Maeve Moran, Geraldine Fitzgerald, David Mockler, Una Geary, Siobhán Corrigan

**Affiliations:** ^1^ Centre for Innovative Human Systems, School of Psychology, Trinity College Dublin, Dublin, Ireland; ^2^ Quality and Safety Improvement Directorate, St James’s Hospital, Dublin, Ireland; ^3^ Inclusion Health Service, St James’s Hospital, Dublin, Ireland; ^4^ Department of Clinical Medicine, School of Medicine, Trinity College Dublin, Dublin, Ireland; ^5^ The Library of Trinity Dublin College, Trinity College Dublin, Dublin, Ireland

**Keywords:** inclusion health, end-user requirements, mobile health record app, integrated care, homelessness, opiate substitution therapy, personal health record, trauma-informed care

## Abstract

**Background:**

Research into mobile health record apps has focused on narrow outcomes, such as medication adherence for persons experiencing chronic conditions. However, no review has examined their use in the context of social exclusion. Persons experiencing social exclusion (PESE) face complex health needs, limited healthcare access, and increased exposure to traumatic life experiences. It is imperative to consider a trauma-informed and integrated care approaches when developing an app for them, and they should be involved as key stakeholders to ensure equitable care. This review examined these apps’ functionalities and features that support PESE in relation to their reported outcomes and the delivery of a trauma-informed and/or integrated care.

**Methods:**

A systematic search of ten databases: Web of Science Core Collection, Medline, PsycINFO, CINAHL, Cochrane, Embase, Scopus, ProQuest Dissertations and Theses A&I, Lenus and OpenGrey International were undertaken, and was supplemented with non-indexed and grey literature. Searches were undertaken in April 2024 in English with no date limit, and used the PRISMA 2020 guidelines. Studies were deemed eligible if they met the SPIDER framework criteria.

**Results:**

One thousand three hundred and thirty-two papers were found eligible for the review, of which eleven qualified for inclusion following screening and quality assessment using QATSDD and MMAT tools. Four themes were found (supporting integrated and connected care; enhancement of user engagement and care coordination; improving data accuracy and access to care; and provision of ongoing monitoring and feedback) related to apps’ functionalities and features, which in turn were linked to reported outcomes. Although a few of these apps’ functionalities and features were aligned with the six principles of trauma-informed care, none of them were implemented considering a trauma-informed care and/or integrated care.

**Conclusion:**

This review provided insights into the complexities of implementing a mobile health record app for PESE. However, limited available data restricted a comprehensive understanding of these apps’ functionalities and features in their specific implementation settings in relation to their reported outcomes. Next steps include translating these findings into survey and interview questions to identify end-user requirements for developing an app for PESE from a trauma-informed perspective to promote integrated care.

**Systematic review registration:**

PROSPERO CRD42024535090.

## 1 Introduction

Social exclusion is defined as a state in which an individual, because of certain characteristics, has markedly reduced access to resources including housing, education, occupation and financial security, and increased exposure to adversity including poverty, homelessness, incarceration, traumatic experiences and stigma (1, 2). Social exclusion is inherently intersectional; individuals frequently experience a combination of overlapping factors that contribute to their marginalisation process, for instance, they may belong to minoritised groups like Black, Traveller, or Roma communities, struggle with severe and enduring mental health or substance use disorders (SUD), and/or face challenges as refugees or as part of the LGBTQI community (3).

Persons experiencing social exclusion (PESE) have much higher rates of ill-health, multimorbidity, early ageing and shorter life expectancy than the general population (4). Difficulty accessing high-quality healthcare is a cardinal feature of social exclusion and is more pronounced in fragmented healthcare systems and where healthcare is privatised (1, 5). PESE frequently experience systemic barriers when trying to access healthcare, which can include social stigma, discrimination, lack of entitlements, and personal barriers, such as self-stigma and a lack of knowledge about available and affordable healthcare options (6–8). This may result in these persons receiving suboptimal medical care.

Trauma-informed care (TIC) is an approach which recognises the impact of traumatic life experiences on individuals and communities and is widely recognised as key to providing equitable care to PESE (9, 10). Central to the philosophy of TIC is acknowledging the effect of historical and current power imbalances and the role this plays in creating health inequities (11). TIC seeks to create safety for PESE by understanding the impact of traumatic life events on health and behaviours (12). Taking a TIC approach focuses on creating safe spaces that minimise potential harm for patients by using the Substance Abuse and Mental Health Services Administration (SAHMSA)'s six TIC principles: safety; trust; collaboration; peer support; empowerment, voice and choice; and awareness of cultural, historical and gender issues (13–15). Any intervention which is developed for PESE should be evaluated against TIC principles to cater to their needs.

A trauma-informed computing framework has been developed to improve technology experiences for PESE (16). The framework enables human-computer interaction (HCI) to embrace TIC to a certain extent, with the aim of minimising harm and reducing the risk of re-traumatisation to enhance user experience, particularly for persons affected by trauma (16, 17). The design of any app's user interface – including both the HCI aspects of task support tools and the information presented to users – is determined by the underlying process design (18). The framework adapts the SAHMSA's six TIC principles to safety, trust, peer support, collaboration, enablement, and intersectionality (16). A trauma-informed perspective to design supports the assessment of functionalities and features by considering how the effects of trauma could be influencing the behaviours of patients and healthcare professionals when using an app (17, 19).

An integrated care approach recognises the complex needs of persons accessing healthcare by addressing both health and social care aspects. The approach aims to address the fragmentation and lack of care coordination often experienced by persons with chronic and complex conditions (20). This approach is vital for effective organisational design and performance (20) and clearly aligns with TIC given that integrated care promotes empowerment of patients and collaboration between services and patients (21), but lacks a universally accepted definition (22). From a patient's perspective, it can be defined as: “I can plan my care with people who work together to understand me and my carer(s), allow me control, and bring together services to achieve the outcomes important to me” (23, 24). The benefits of integrated care include fewer patient appointments, better continuity of care, better coordination of services, more personalised care, reduced cost, improved quality of life and safety (25). This improves healthcare processes in a coordinated manner to promote care continuity.

The International Foundation for Integrated Care indicates that the digital solutions pillar is one of the nine pillars of integrated care (26). Digital solutions, such as electronic health records (EHR), can be considered integral parts that connect various building blocks in healthcare to support integrated care (27). These technologies serve as a unifying mechanism by ensuring a flow of information in the healthcare system (28, 29), but this vital role of these technologies can be compromised if healthcare professionals, for instance, find the EHR to be disorganised or overly complex to use, potentially leading to adverse health outcomes (30). Overall, research has found that regular use of electronic records can lead to better patient care and safety (31), improved organisational efficiency (32, 33), and promote integrated care (27).

Recent years have seen the development of personal health record (PHR) systems (34), such as the My Health Record (MyHR) system (35). In this study, the term PHR refers to “an electronic application through which individuals can access, manage, and share their health information, and that of others for whom they are authorised, in a private, secure, and confidential environment” (36). This comprehensive definition was provided in a personal health working group final report by the Connecting for Health collaborative, a public-private effort led by the Markle Foundation to develop interoperable health information infrastructures (37), and is supported by standards from organisations like the International Organisation for Standardisation (38).

The primary advantage of PHR lies in the patients’ capacity to manage their own health information (39, 40). Nonetheless, numerous obstacles must be addressed to facilitate the widespread adoption of PHR, such as achieving interoperability with EHR systems and addressing privacy and security issues that may arise from their use (41). Even though the term PHR can denote records in various formats, including paper, they are usually implemented electronically and can be accessed through mobile devices (39). For this study, the term “*mobile health record app*” is used to reflect a mobile and web-based application that can serve as a digital repository for a patient's health data, which can be controlled and managed by a patient or authorised representative (38, 42, 43).

Even with these advances in healthcare, there is still a critical issue: PESE who often have the highest care needs, continue to have the worst access to healthcare services. This disparity illustrates Tudor Hart's Inverse Care Law, which posits that those most in need of healthcare are least likely to receive it (44). The leverage of the ongoing evolution of technological advances such as electronic record-keeping, mobile health record app presents a promising opportunity to address this gap. A mobile health record app, which is a PHR and EHR, has the potential to promote health and social care coordination and planning, to increase patient safety, control and empowerment and to redress power imbalances. However, it is crucial to understand the end-user requirements for such an app from a trauma-informed design approach, which aims to minimise harm and re-traumatisation while improving user experiences, particularly for persons affected by trauma (16, 17, 19). Otherwise, such an app (as well as other elements of technological progress) runs the risk of inadvertently reducing access to healthcare for PESE and/or increasing levels of disempowerment and thereby worsening health inequalities and retraumatising PESE.

Systematic literature reviews (SLRs) on mobile health record apps have been published (34, 35, 39, 45–48), but there has been no review to date to look at the use of such apps in the context of social exclusion, traumatic life experiences and health inequalities. Mobile health record apps are complex interventions whose effectiveness is influenced by the context in which they are used. The aim of this study was to understand which mobile health record apps support PESE. The primary objective of this review was to assess the functionalities and features of mobile health record apps that support PESE in relation to their reported outcomes and the delivery of trauma-informed and/or integrated healthcare. The secondary research objectives included (i) identifying the main users of the apps and describing their recruitment methods, and (ii) assessing the key features of the apps, with particular attention to their multiplatform accessibility and interoperability within healthcare systems.

## 2 Methods

### 2.1 Defining the review scope

#### 2.1.1 Protocol and registration

This review adhered to Preferred Reporting Items for Systematic Reviews and Meta-analyses (PRISMA) 2020 guidelines (49). The protocol has been registered with PROSPERO under registration number CRD42024535090.

#### 2.1.2 Eligibility criteria

The research team members (CNC, MEW and FI) outlined the inclusion and exclusion criteria as per the Sample, Phenomenon of Interest, Design, Evaluation, Research type (SPIDER) framework (50). The SPIDER question framework is highly effective for qualitative or mixed methods research topics focused on samples rather than populations (50). Only intervention-based studies were included so therefore survey-based studies were excluded. Studies were deemed eligible if they met the SPIDER framework criteria as outlined in Table 1. Papers were screened for eligibility for inclusion by the three co-authors, of whom two, CNC and MEW, are senior academics with experience in conducting SLRs, to define the eligibility criteria. There were no restrictions on geography or publication year if the study was in English for practical reasons.

**Table 1:** Eligibility criteria.

Element	Definition	Inclusion criteria	Exclusion criteria
Sample	Who is the group of people being studied?	Studies that focus on persons experiencing social exclusion, such as homelessness, drug dependence, refugees and indigenous populations.	
Phenomenon of interest	What are the reasons for behaviour and decisions?	Studies that focus on the health information systems that contain the use of a mobile health record app to support individuals experiencing social exclusion.	Studies focusing on m-health intervention, mobile app intervention, ehealth intervention, digital intervention, or short message system (SMS) intervention for persons experiencing social exclusion.
Design	How has the research been collected (e.g., interview, survey)?	Focus groups, questionnaires, interviews, observations, experiments, cases and document reviews.	Survey based study only.
Research type	What type of research (mixed methods)?	Intervention-based studies including qualitative, quantitative or mixed methods.	Feasibility study only.
Evaluation	What is the outcome being impacted?	Improve outcomes for the patients, staff members, and service system for integrated and trauma-informed care.	

### 2.2 Search strategy

A systematic electronic search of the English-language literature, with no date limits, was conducted across ten electronic bibliographic databases recognised for their relevance to technology and health research between April 1 and April 29, 2024. Seven bibliographic databases; Embase.com (date of inception 1971), Medline ALL via Ovid (1946 to Daily Update), Web of Science Core Collection, SCOPUS, CENTRAL trial registry and PsycINFO/CINAHL via EBSCOhost, along with three grey literature databases; ProQuest Dissertations and Theses A&I, Lenus, and OpenGrey International, were searched. Search terms were developed based on the research question to identify mobile health record apps supporting socially excluded individuals. The search contained terms for (1) socially excluded people, (2) mobile applications, and (3) electronic health records. Terms were combined with the Boolean operators (AND, OR) and proximity operators to form search phrases. The process was conducted with the support from a subject matter librarian and a co-author (GF). The searches were refined by using relevant thesaurus terms from Emtree for Embase and Medical Subject Headings (MeSH) for Medline, with additional support from another subject matter librarian and co-author (DM). These searches were then adapted to other bibliographic databases. Detailed search strategies for Medline, Embase, and other bibliographic databases are provided in Supplementary File S1, with a sample search strategy for Web of Science Core Collection shown in Table 2. Additional publications were also identified through manual search and consultation with two senior international academics in the field to capture non-indexed and grey literature.

**Table 2:** A sample of search strategy and combination of keywords for Web of Science Core Collection.

Search string	Key words
1	(“mobile health” OR mHealth OR m-Health) OR (Smartphone* NEAR/3 app*)
2	[(Medical OR health OR virtual OR electronic) NEAR/3 record*] OR (“medical history” OR e-record OR EMR OR EMRs OR EHR OR EHRs)
3	[Social* NEAR/2 (justice OR exclusion OR excluded OR marginalised OR marginalized OR disadvantage* OR inclusi* OR discriminat*)] OR (“diversity equity and inclusion” OR underserved OR “multiple disadvantages” OR “hidden disabilit*”) OR [Health NEAR/1 (disparit* OR equit* OR inequit* OR inequalit*)] OR homeless* OR (Rough NEAR/3 sleep*) OR [(Insecur* OR inadequate OR precarious OR instability) NEAR/3 (housing OR accommodation)] OR [(displaced or undocumented) NEAR/3 (person* OR people OR immigrant* OR population*)] OR “forced migrant*” OR “migrant worker*” OR “forced migration*” OR refugee* OR “asylum seeker*” OR “unaccompanied minor*” OR addict* OR alcoholi* OR [(substance OR drug) NEAR/1 (abuse* OR use*)]
4	#1 AND #2 AND #3

### 2.3 Choosing and appraising the evidence

#### 2.3.1 Choosing the evidence

All papers were imported into Covidence software to support the screening process (51). Papers were independently screened by title and abstract for inclusion or exclusion by two members of the research team (MM and FI). All conflicts were resolved without the involvement of a third person. The papers which passed the aforementioned stage were subjected to full-text review once more independently by members of the research team working in pairs (MEW and CNC) and (FI and SC). The authors deliberated on each paper's eligibility criteria during both screenings and resolved any conflicts without the involvement of a third person.

#### 2.3.2 Appraising the evidence

The selected tool for quality appraisal was the Quality Assessment Tool for Studies with Diverse Designs (QATSDD) (52). The QATSDD is a 16-item quality assessment tool that was created to assess research that integrates various study designs and has been used for the appraisal of mixed-methods studies only [e.g., (53)]. The tool contains 16 criteria questions that are scored on a Likert scale ranging from 0 to 3, with a score range of 0 to 48. The QATSDD appraisal tool was complemented by the Mixed Methods Appraisal Tool (MMAT) in order to facilitate the evaluation of studies that incorporate mixed studies (qualitative, quantitative, and mixed methods studies) (54, 55). The MMAT enables the concurrent evaluation and description of the methodological quality of three methodological domains: mixed, qualitative, and quantitative (also subdivided into three subdomains: randomised controlled, non-randomised, and descriptive) (54, 55).

### 2.4 Extracting and synthesising data

The process used for data extraction was important in terms of its completeness, and the research team reviewed data extraction to make sure it was appropriate. The extraction table was developed by the primary author (FI) and was improved after discussion with a senior author (MEW). Specific information about each mobile health record app was gathered, including details about the app's main features, interoperability, multiplatform capabilities and the setting in which it was implemented. Furthermore, contextual information regarding user types and recruitment processes were collected to understand factors that facilitated or hindered implementation and their resultant outcomes. This supported the data analysis, which resulted in four themes that provided a clearer representation of functionalities and features that contributed to the observed results across the studies.

## 3 Results

### 3.1 Evidence selected and quality assessment

**Figure 1:**
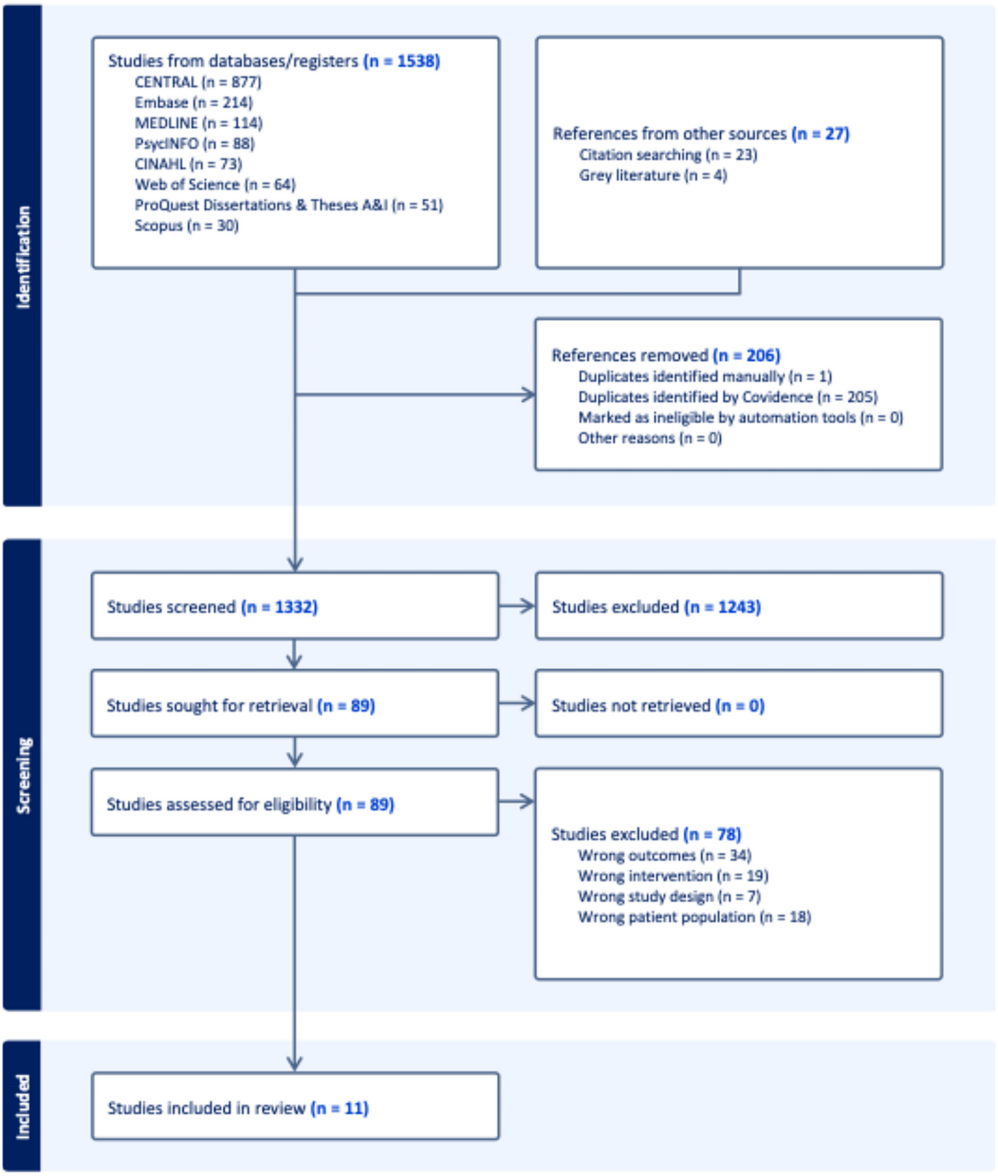
PRISMA flow diagram.

One thousand three hundred and thirty-two papers were found eligible for the SLR, of which eleven qualified for inclusion following quality assessment and screening. Please see the PRISMA Flow Diagram (Figure 1) for more details. The range of scores for the studies from the QATSDD was between 24 and 38 with a mean score of 28, which is in a good quality range (52). This indicates that studies with this mean score are at a relatively low risk of bias (52). The papers were evaluated by the primary author, and the list of papers with their evaluation scores is available in Supplementary File S2. MMAT appraisal tool was used to supplement QATSDD tool in terms of appraising methodologies of included studies. The calculation of an overall score from the ratings of each criterion is not recommended (54). Instead, it is recommended that a more comprehensive presentation of the ratings of each criterion be provided to enhance the quality of the studies included (54). The ratings for MMAT can be found in Supplementary File S3.

### 3.2 Key study characteristics

Table 3 depicts key characteristics of included studies. This review included studies conducted in Bangladesh (*N* = 1) (56), the United States of America (*N* = 4) (57–60), Pakistan and Afghanistan (*N* = 1) (61), Kenya (*N* = 1) (62), Italy (*N* = 1) (63), China (*N* = 1) (64), Lebanon and Jordan (*N* = 1) (65), and Canada (*N* = 1) (66). Most studies were conducted in a primary healthcare setting (*N* = 7) (56–61, 66), followed by humanitarian setting (*N* = 3) (62, 63, 65), and community-based setting (*N* = 1) (64).

**Table 3:** Key study characteristics.

Reference	Location	Sample (Who is the group of people being studied?)	Phenomenon of Interest (What are the reasons for behaviour and decisions?)	Design (How has the research been collected (e.g., interview, survey?)	Research type (What type of research (mixed methods?)	Evaluation (What is the outcome being impacted?)
(65)	Lebanon and Jordan	Refugees and their healthcare professionals	An open-source electronic health record (EHR) system – Hikma app – designed to meet the healthcare needs of displaced populations in low-resource settings	Focus Group and Interview	Intervention-based mixed-method study	The healthcare delivery for refugees, particularly in terms of clinical efficacy, organisation, and planning of healthcare services, and ability to maintain patient records
(63)	Italy	Migrants	A mobile health system – PANDA – to improve antenatal care for vulnerable pregnant women seeking asylum	Questionnaire and system usage data report	Intervention-based quantitative study	Quality and comprehensiveness of antenatal care provided to migrant women in the largest reception centre in Europe
(60)	United States	Healthcare professionals involved in caring for persons suffering from HIV and drug addiction	A mobile platform for care coordination intervention (CCI) and its assessment procedures for persons experiencing HIV and substance use disorders.	Interview and functionality testing with healthcare professionals	Intervention-based mixed-method study	Quality and frequency of interagency communication, patient retention in dual care, and relational coordination among healthcare professionals supporting persons experiencing HIV and drug addiction
(66)	Canada	Underserved population	An exercise prescription app that could be integrated into the clinic's electronic health record (EHR) system for underserved patients	Feedback through a quality improvement approach (Plan-Do-Study-Act cycle)	Intervention-based qualitative study	The prescription and adherence to individualised exercise programs for patients with musculoskeletal disorders in primary care
(62)	Kenya	Refugees	A mobile app – Sana – designed to manage non-communicable diseases (NCDs) such as hypertension and diabetes in a humanitarian context	Questionnaire and Interview	Intervention-based mixed-method study	The continuity, quality, and efficiency of chronic disease care
(56)	Bangladesh	Underserved rural patients and their healthcare professionals	A mobile clinical decision support system and healthcare information dissemination platform for an underserved rural population	Questionnaire and Focus Group	Intervention-based mixed-method study	Healthcare equity and patient engagement among underserved populations
(57)	United States.	Persons experiencing chronic conditions	A bidirectional text messaging with clinical information systems and electronic medical records (EMR) for enhancing chronic disease management for underserved patients	Focus Group and system usage data report	Intervention-based mixed-method study	Patient engagement in self-management behaviours and health information awareness about chronic disease care
(58)	United States.	Persons experiencing drug addiction	A mobile health record app – Seva – designed to support patients suffering from alcohol use disorders.	Use of RE-AIM framework and collection of system usage data	Intervention-based quantitative study	The implementation outcomes (feasibility, adoption and sustainability of a mobile system in primary care settings) and clinical effectiveness outcomes (patient engagement, retention in treatment and potential improvement in substance use disorder outcomes)
(64)	China.	Persons experiencing drug addiction	A community-based addiction rehabilitation electronic system (CAREs) designed to assist persons using drugs and their social workers	Randomised Control Trial	Intervention-based quantitative study	The feasibility of CAREs using overall proportion and frequency of features used in both app and webpage end, and effectiveness using percentage of drug-positive samples, longest period of abstinence, contact times with social workers, and the change in addiction severity
(61)	Pakistan and Afghanistan.	Community health workers and female health workers in remote areas	A mobile app – Hayat – designed to digitalize and facilitate electronic record-keeping of maternal and childcare services in remote areas for community health workers in Pakistan and female health workers in Afghanistan	Focus Group	Intervention-based qualitative exploratory study	Efficiency and effectiveness of primary care service delivery and work satisfaction among community health workers in Pakistan and female health workers in Afghanistan
(59)	United States.	Underserved populations	A health information technology platform – imHealthy app – designed to assess and improve the well-being of persons in medically underserved communities	Focus Group	Intervention-based qualitative study	The well-being measures such as quality of life and resilience of underserved populations

**Figure 2:**
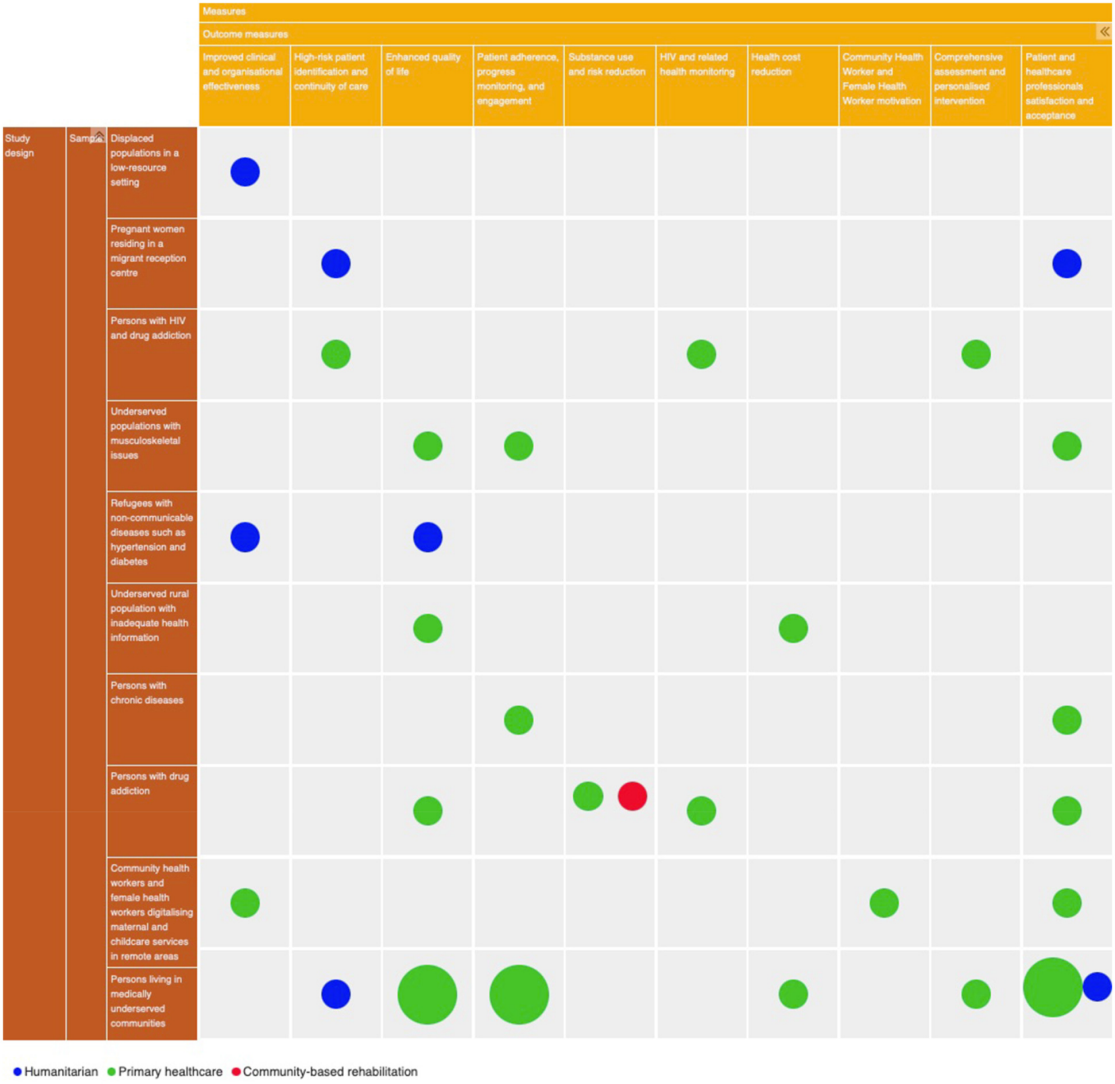
Evidence map.

None of the eleven studies pertaining to the implementation of the mobile health record apps focused on persons experiencing homelessness or indigenous populations. The map of evidence is shown below as Figure 2 to demonstrate where there are gaps and evidence clusters. A large number of studies focused on developing mobile health records infrastructure for digitalising health records of PESE in their respective healthcare settings (56, 59, 65) and drug dependence (58, 60, 64). A few numbers of studies focused on maternal and childcare services (61, 63), chronic disease like diabetes (57, 62), and musculoskeletal conditions in the context of social exclusion (66).

### 3.3 Primary research question

**Figure 3:**
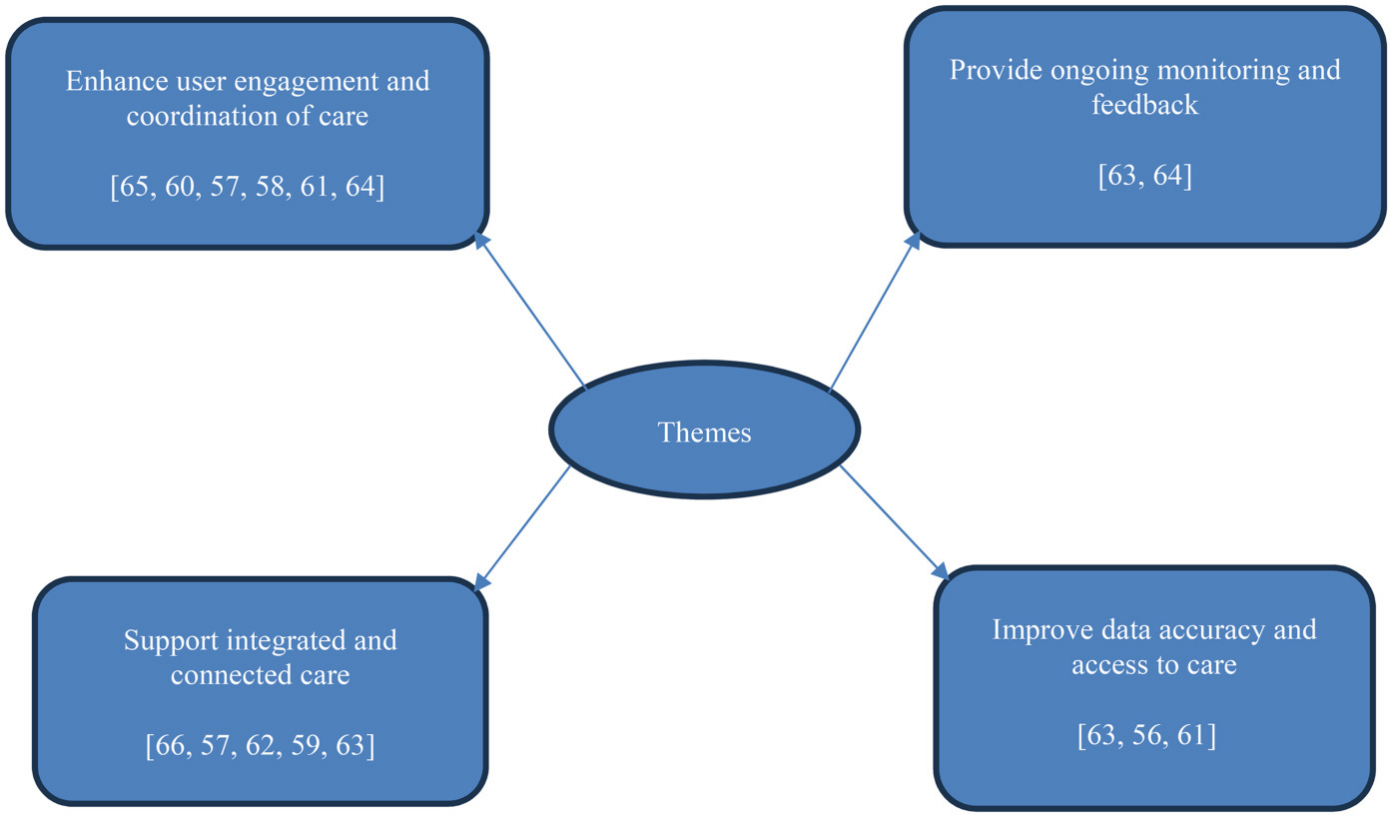
Themes from the synthesis of included studies.

The primary objective of this review was to assess the functionalities and features of mobile health record apps that support PESE in relation to their reported outcomes and the delivery of trauma-informed and/or integrated healthcare. A thematic map depicted in Figure 3 indicates four themes that emerged from the literature review, including:

(i) Theme 1 Enhancement of user engagement and coordination of care; includes functionalities and features, such as provision of tools for instantaneous health data generation, appointment reminders, multilingual support with real-time translation into the user's preferred language and offline functionality, that would improve continuity of care and access across healthcare services and systems, particularly in supporting PESE.
(ii) Theme 2 Supporting integrated and connected care; involves functionalities and features, such as the capability of synchronising with existing health information technology systems, combined with secure documentation and standardised access to patient records, that would enable healthcare professionals to leverage longitudinal patient data for decision-making, to address the issue of healthcare services fragmentation often caused by siloed systems.
(iii) Theme 3 Emproving data accuracy and access to care; includes functionalities and features, such as the customisation of app features to align with patients’ specific needs and supporting patients with recording their questions and the responses of healthcare professionals, that would improve health information quality and access to personalised care.
(iv) Theme 4 Provision of ongoing monitoring and feedback; involves functionalities and features, such as being accessible to healthcare professionals on both smartphones and desktop web browsers in relation to wireless transmission of health records securely and provision of real time feedback and support, that enhance standard of care and monitoring of patients.

The most commonly cited theme across the studies was the enhancement of user engagement and coordination of care, which was mentioned in six studies (57, 58, 60, 61, 64, 65). This was followed by supporting integrated and connected care, which appeared in five studies (57, 59, 62, 63, 66). Improving data accuracy and access to care was the next most frequently cited theme, which was mentioned in three studies (56, 61, 63). Lastly, the provision of ongoing monitoring and feedback was the least cited theme, which appeared in two studies (63, 64). These findings are presented in Table 4 which facilitated clearer representation of the complex interplay of functionalities and features of mobile health record app that contribute to the resultant outcomes across eleven studies in their respective implementation healthcare settings.

**Table 4:** Functionalities and features of Mobile health record apps that support PESE in relation to their reported outcomes.

Theme	Study	Setting	Functionalities and features	Outcome
1. Enhance user engagement and coordination of care	(57, 58, 60, 61, 64, 65)	Mobile health record apps which were implemented in primary or humanitarian healthcare settings	• provides patients with continuous monitoring of the status of their health conditions • provides patients with recurring reminders to enter health records • provides patients with tools for instantaneous data creation • can provide patients and healthcare professionals with offline functionality • can provide patients and healthcare professionals with multilingual interfaces • facilitates patient peer support in encouraging treatment adherence	• a reduction in the healthcare system strain • improving care coordination across settings • enhancing patient experience • facilitating follow-up care regardless of location or time
2. Support integrated and connected care	(57, 59, 62, 63, 66)	Mobile health record apps which were implemented in primary or humanitarian settings	• can synchronise with existing health information technology systems like the Electronic Health Record system • allows for secure documentation and access to standardised patient records • supports patients with individual treatment plans • empowers healthcare professionals with longitudinal patient information for decision making • provides patients with access to comprehensive health information from multiple healthcare professionals	• improved disease management • better integration of services • improved retention in care • enhanced health outcomes for patients with complex conditions
3. Improve data accuracy and access to care	(56, 61, 63)	Mobile health record apps which were implemented in humanitarian or rural healthcare settings	• can be customised for personalised care • facilitates better data management • reduce missing and erroneous entries and make data more transparent and accountable • supports communication between healthcare professionals by exchanging images and voice notes • can help patients ask questions about health conditions and medicines and receive answers from healthcare professionals	improvement in • care continuity • access to health services • the quality of health information for underserved populations
4. Provide ongoing monitoring and feedback	(63, 64)	Mobile health record apps which were implemented in a community-based or humanitarian healthcare settings	• can transmit health records wirelessly • can provide healthcare professionals with alerts for abnormal clinical results • can provide patients with real-time feedback and support • improves user tracking • allows for identifying high-risk patient behaviours	• enhancement in the standard of care • a reduction in care duplication • a reduction in the likelihood of adverse events arising from user behaviours

None of the eleven papers pertaining to the implementation of a mobile health record app within healthcare settings considered their use from a TIC perspective or focused specifically on the impact of the mobile health record app on integration of care. One study noted that the app implementation team worked in an integrated care model (66). The apps' functionalities and features were mapped out against six principles of the trauma-informed computing framework, as illustrated in Table 5. While a few functionalities and features corresponded to each of the six principles of the framework, there were no examples related to the *Safety and Trust* principles – apart from allowing users to access secure documentation and standardised patient records. This feature is consistent with one Safety principle aspect, which emphasises the importance of safeguarding users from digital hazards, including malicious software and unauthorised access (16). There was no element that was consistent with the other aspect of the Safety principle, psychological safety, in relation to the usage or navigation of these apps (16). In addition, there was no feature and functionality that falls into the Trust principle. Safety and Trust principles are closely connected; trustworthy computing requires that technology, processes, and organisations function transparently, predictably, and reliably, while also enabling users to learn from errors and make corrections as needed (16, 67).

**Table 5:** A mapping of functionalities and features against six trauma-informed computing principles.

Six trauma-informed computing principles	Study	Functionalities and features
Safety	(57, 62, 63)	• allowing users to access secure documentation and standardised patient records.
Trust		Trust wasn't explicitly highlighted in the included studies
Peer support	(58)	• facilitates patient peer support in encouraging treatment adherence
Collaboration	(56, 61, 64)	• provides patients with continuous monitoring of the status of their health conditions • can help patients ask questions about health conditions and medicines and receive answers from healthcare professionals
Enablement	(57, 60, 61, 64)	• provides patients with access to comprehensive health information from multiple healthcare professionals • provides patients with recurring reminders to enter health records • provides patients with tools for instantaneous data creation • can provide patients with offline functionality • can provide patients with real-time feedback and support
Intersectionality	(62, 65, 66)	• can be customised to each patient's needs for personalised care • can provide patients and healthcare professionals with multilingual interfaces • supports patients with individual treatment plans

### 3.4 Secondary research questions

The secondary research objectives included (i) identifying the main users of the apps and describing their recruitment methods, and (ii) assessing the key features of the apps, with particular attention to their multiplatform accessibility and interoperability within healthcare systems. These questions will now be addressed.

#### 3.4.1 Mobile health record app main users and their recruitment process

Table 6 below presents the types of users involved in the eleven studies and their recruitment methods. In most studies, there were two types of users for the mobile health record app: PESE served as the primary users in the majority of cases (56–58, 64), and healthcare professionals as secondary users (56–59, 63). Fewer studies have reversed these roles, with healthcare professionals (including community health workers and female health workers) as primary users (60–63, 65, 66), and PESE as secondary users (66). Additionally, a smaller number of studies included social workers as either primary (59) or secondary users (64).

**Table 6:** App main users and their recruitment methods.

Study	User	User recruitment
(65)	The main users were the healthcare professionals, such as, doctors, nurses and administrative staff in their practice in humanitarian settings in Lebanon and Jordan.	The study used existing staff members at the healthcare settings in Jordan and Lebanon.
(63)	The primary users were Community Health Workers digitalising health records of pregnant women residing in the largest European migrant reception centre in Sicily, Italy. The secondary users were healthcare professionals such as physicians responsible for medical unit and checking completeness of data.	Not stated.
(60)	The users were healthcare professionals for PESE experiencing Human Immunodeficiency Virus (HIV) and substance use disorder.	The study used existing staff members at Northeast region of the United States.
(66)	The primary users were healthcare professionals such as physicians, nurses, physiotherapists, and chiropractors. The secondary users were patients, with low social economic status, receiving musculoskeletal exercise rehabilitation through the app.	The used existing staff members and patients from the academic family medicine clinic in Canada
(62)	Healthcare professionals such as General Practitioners, nurses, and other clinical staff using a mobile health record app in a humanitarian setting in Kenya.	The study used existing staff members at the International Rescue Committee (IRC) health facilities in the Hagadera refugee camp.
(56)	The primary users were rural residents experiencing poverty, limited educational opportunities, and low health literacy. The secondary users were healthcare professionals, such as General Practitioners, consulting patients virtually and offering diagnosis or treatment based on queries asked and information provided by the residents to them in Bangladesh.	No details on the recruitment process. But the study implied that the project team engaged in outreach efforts to reach potential users.
(57)	The primary users were underserved patients who were part of the chronic disease such as diabetes registry at Denver Health Clinic. The secondary users were healthcare professionals supporting patients through a mobile health record app with automated and bidirectional message.	Users were current patients on the diabetes registry at the Denver Health Clinic.
(58)	The primary users were patients suffering from substance use disorder. The secondary users were healthcare professionals primarily through a web portal that provided them with longitudinal information about their patients’ substance use and well-being.	Eligible patients were recruited by healthcare professionals at the healthcare facility.
(64)	The primary users were individuals living with substance use disorders. The secondary users were social workers providing service and monitoring drug use behaviour	Drug users were recruited through a Social Worker station in Shanghai, China.
(61)	The users were Community Health Workers and Lady Health Workers facilitating the digitalisation of patients ‘health records.	The study was nested within a larger quasi-experimental study assessing the effectiveness of a mobile health record app for improving maternal and child health in Pakistan and Afghanistan.
(59)	The primary users were social workers supporting patients without health insurance coverage, with financial or linguistic difficulties or lacking a primary doctor p in a free clinic in Pittsburgh, Pennsylvania. The secondary users were the clinic administrator to associate unique identities with the corresponding participants.	The study used existing staff members at the Pittsburgh Free Health Centre.

Most studies used existing channels within their respective healthcare settings to recruit staff members and patients to participate in the studies (57–62, 64–66), while a few studies did not specify their recruitment methods (56, 63).

#### 3.4.2 Mobile health record app multiplatform accessibility and interoperability within healthcare systems

Table 7 illustrates the interoperability and multi-platform accessibility of mobile health record apps. Most studies indicated that the apps supported integration with existing health information technology systems (56, 60, 61, 66, 68); though fewer were integrated with existing systems in their respective implementation context (59, 62), while one had a wireless transmission functionality (63). A limited number of studies have not addressed this feature (64, 65), and one study explicitly stated that the app lacked integration capability (58). Regarding platform accessibility, most studies did not specify iOS and Android compatibility (56–62, 64, 65). While a significant number described the apps as mobile device-compatible (56, 58, 62, 64, 65), fewer specified web-based and mobile app functionality (57, 61), and others mentioned tablet compatibility (59, 60). One study noted that the app worked on Android smartphones and was available in English and French (63).

**Table 7:** App multiplatform accessibility and interoperability within healthcare systems.

Study	App interoperability	App multi-platform accessibility
(65)	Not stated.	The app was designed as a mobile app and it's unclear whether it accessible on iOS or Android platforms.
(63)	The mobile health record app could facilitate the creation of electronic patient records and their automatic wireless transmission to existing health information technology systems.	The app was compatible with Android smartphones and available in English and French.
(60)	The mobile health record app was designed with the potential for future integration with existing Electronic Health Record systems.	The mobile app was designed to be used on tablet devices. It is unclear if it is a web-based mobile app or a native app for iOS or Android platforms.
(66)	The mobile health record app was designed to support integration with existing health information technology systems, specifically the clinic's Electronic Health Record system.	It was designed for Personal Computers (PCs) and was incompatible with the iOS and Android platforms.
(62)	The mobile health record app was integrated with existing health information technology systems such as Electronic Medical Records and CommCare platform for better data management and reporting.	It was designed primarily for use on mobile devices, and the study did not provide details regarding its availability on iOS or Android platforms.
(56)	It was not stated, but the study indicated that the mobile health record app prototype had a mobile front-end for data distribution and a synchronisation mechanism which can enable integration with other health information technology systems.	The study discussed the design for mobile and handheld devices, and it's unclear whether the app was accessible on iOS and Android platforms.
(57)	The mobile health record app supported integration with existing health technology information systems like the Electronic Medical Records and facilitated data sharing between services.	The study does not specify whether the app was a web-based and mobile app or whether it was specifically designed for iOS or Android platforms.
(58)	The mobile health record app was not successfully integrated into the existing Electronic Health Records at the clinics involved in the study because of interoperability issues.	The app was described as a mobile health system, which usually means that it is designed for use on mobile devices. It is unclear whether the platform was available for iOS or Android.
(64)	Not stated.	The app was designed as a smartphone app, but the study does not specify whether it was available on iOS, Android platforms.
(61)	The mobile health record app was designed to support integration with existing health information technology systems.	It was a web-based and mobile app and comprised of two components: a smartphone app for data entry and a web dashboard for healthcare professionals. It's unclear whether the app was available iOS and Android operating systems.
(59)	The mobile health record app included a custom-designed Electronic Health Record that was integrated with the mobile app.	The mobile app was distributed on mobile devices (tablets) to Social Workers. It's unclear whether the mobile app was available for iOS or Android platforms.

## 4 Discussion

This SLR is the first to specifically focus on identifying the functionalities and features of mobile health record apps implemented in healthcare settings supporting PESE to promote integrated, trauma-informed care. The SLR facilitated an understanding of the use of these apps in various healthcare settings through a systematic and transparent review of the relevant literature. None of the eleven studies on the implementation of mobile health record apps considered their alignment with the principles of TIC. The study contributes to the existing literature because no previous study has focused on which mobile health record apps support the PESE until now.

This study builds upon previous SLRs of mobile health record apps (34, 35, 39, 45–48). Four key themes: health information fragmentation, health information quality and access, adverse medical events and duplication of care, and care coordination, were identified in the SLR conducted on the use of PHR in the general Australian population (35). Their findings revealed mixed outcomes regarding the success of PHR implementation in Australia. For instance, the study indicated that the PHR system has the potential to decrease fragmentation of health information; however, challenges related to workforce adoption were recognised as an issue (35). In our study, we also found four themes, with enhancement of user engagement and coordination of care as the most cited theme. This is important as mobile health record apps can be designed and implemented to enable integrated care across different healthcare settings that are involved in caring for the persons irrespective of geographical location and time.

Contradictory results in relation to supporting coordination of care were found (35). However, our study found that (as per Theme 1), a mobile health record app can enhance engagement of users; either patients or healthcare professionals, and improve the overall coordination of care, especially for PESE who may experience challenges in accessing and using healthcare services in primary, humanitarian, or community-based healthcare settings. For instance, our study findings indicated that such an app would empower patients by providing tools for instantaneous health data generation, appointment reminders, multilingual support with real-time translation into the user's preferred language, and offline functionality to enhance care continuity access across healthcare services and systems, particularly in supporting PESE (58, 60, 61, 64, 65). The offline functionality means that users may use it without needing constant internet connectivity, which may be particularly important in humanitarian settings or for patients who lack access to the internet (61, 65). The functionality for creating data, for example, would enable patients to create summary care records and scan of documents that may be needed for different services e.g., identity details, and proof of payment and address, which could enhance communication and coordination across healthcare settings. The SLR on PHR taxonomy and their challenges compliments this by indicating that photos and scanned documents are the data types that are included in the PHR system (39), and would be critical for the continuity of care across time and space for PESE (45). In addition, the multilingual support and instantaneous translation to one's mother tongue feature signifies that the mobile health record app could cater to the needs of PESE, such as refugees, who may have difficulties with the language of the country in which they find themselves (65). This is further support by the SLR on the important design features of PHRs to improve medication adherence for persons experiencing chronic conditions, which highlighted design features in mobile health record apps – such as reminders, medical appointment management, diaries, and self-monitoring – that improve medication adherence for patients with chronic conditions (34).

The study offered insights into the complexities of using a mobile health record app for PESE and could help in understanding the dynamics that could contribute towards success or failure of such apps in healthcare systems. Our findings indicated that a mobile health record app could address healthcare fragmentation across different health settings for PESE (as per Theme 2). These study findings are supported by results of the SLR on PHR in the general Australian population, which, despite highlighting challenges in adoption by both patients and healthcare professionals, stressed its potential benefits (35) and are complemented by the findings of the SLR on PHR, which demonstrated that participants attributed a high level of value to this technology in relation to the co-location, viewing, updating, and sharing of health information with clinicians (69). Our findings suggested that, for example, implementing a mobile health record app capable of synchronising with existing health information technology systems, combined with secure documentation and standardised access to patient records, would enable healthcare professionals to leverage longitudinal patient data for decision-making, to address the issue of healthcare services fragmentation often caused by siloed systems (59, 60, 62, 63). The synchronisation capability, which would enable health data sharing across multiple health information technology systems, could address the interoperability challenge (39), that hindered the effective implementation of mobile health record apps in their respective clinical settings, e.g., (58). In a study on practical and ethical issues for patients and physicians using PHR, the results suggested that a PHR that can efficiently integrate information from multiple sources may save time and healthcare costs by providing a useful summary, but physicians may not be able to use the data if it contains data that were generated by a patient, which can be susceptible to incomplete, inaccurate, and outdated information (43). A functionality for standardising patient record with clear representation of user-entered information and an intuitive display for healthcare professionals would be an important step towards care quality and continuity (57). Furthermore, a functionality for empowering healthcare professionals with longitudinal health records (e.g., blood pressure measurements) would strengthen decision-making in chronic disease management (58, 62). This is illustrated in the SLR, which assesses the functionality and utility of PHR in the clinical context of record-keeping health information, such as medical history, medications, laboratory tests, and vaccinations (46).

This SLR highlighted that a mobile health record app could improve data accuracy and access to care (as per Theme 3). The results of the SLR on the use of PHR in the general Australian population supports this finding by reporting improvements in health information access, despite issues related to data reliability and completeness (35). Our study findings demonstrated that, for instance, the customisation of app features to align with patients' specific needs and a functionality to support patients with recording their questions and the responses of healthcare professionals would improve health information quality and access to personalised care (56, 61, 63). The customisation feature would mean that, for example, there could be a “read-only” access option in the app for essential documents such as prescriptions and vaccination records. This opportunity for customisation would be possible due to the interactive interface of the mobile health record app (48). Recording patient questions and responses from healthcare professionals would address shared decision-making (SDM) in managing for PESE's health conditions (56). Recording a patient's questions was reported as one of the features of a mobile health record app in the SLR on the attributes of PHR for patients with multiple sclerosis (47). This would help with health information management and facilitate administrative reporting. It would also facilitate SDM and improve overall condition management. SDM involves the healthcare professional and the person working collaboratively to make informed decisions about the person's care (70, 71).

The study found that, despite previous limited research support, the mobile health record app could still play a significant role in supporting ongoing monitoring and feedback, as outlined in Theme 4. The findings of this study demonstrated that, for example, wireless health records transmission and integrated alert functionalities for abnormal clinical results have enabled healthcare professionals in reducing the likelihood of patient safety events in a community-based and humanitarian setting (63, 64). An app that could be accessible to healthcare professionals on both smartphones and desktop web browsers in relation to wireless transmission of health records securely would be another beneficial feature in relation to ongoing monitoring of patients. This is corroborated by the findings of a study on the types and sources of diagnostic errors in primary care settings, which demonstrated that accessing patients' health records helped healthcare professionals like General Practitioners (GPs) make better decisions and prevent patient safety events like drug interactions (31). An integrated alert function for patients, such as PESE, to contact healthcare professionals for support would be a key feature for the app. This is reflected in the SLR on the important design features of PHRs to improve medication adherence for persons facing chronic conditions, which identified specific design features in mobile health record apps like feedback and alerts and health condition management in mobile health records which contribute to improved medication adherence among patients with chronic illnesses (34).

This study does have some limitations. There was a lack of detailed information in the literature regarding the implementation of mobile health record apps for PESE in their respective healthcare settings. Only eleven papers met the criteria for final synthesis, despite an initial high number of potential studies (one thousand three hundred and thirty-two) identified through database searches and grey literature. This is partially because the field for designing mobile health record app from a TIC perspective to promote integrated care is relatively new (16, 17, 19). There are limitations inherent in the included studies. These selected studies often lacked important information: eight didn't specify the project team's expertise and composition (56, 58, 61–65), four omitted a guiding theoretical framework (57, 59, 62, 64), one was unclear about user recruitment methods (63), two didn't provide details about app interoperability (64, 65), and nine were unclear about multi-platform accessibility (iOS and/or Android compatibility) (56–61, 64, 65). These information gaps limited the available data for synthesis and impacted a comprehensive understanding of functionalities and features of these mobile health record apps in relation to their reported outcomes in their respective implementation contexts. This, in turn, had an impact on the study's capacity to draw conclusions and provide insights into the usage of mobile health record apps that support PESE.

As with most research, the included studies had certain methodological limitations. Two lacked standardised outcome measures (65, 66), one had limited outcome measures (63), one had unclear evaluation criteria and outcome measures (56), and one lacked quantitative outcomes (57), three studies had a potential bias due to its reliance on self-reported data (60, 63, 65), five had a potential selection bias (56–58, 61, 64), and one had a non-random recruitment (66). Furthermore, six studies had a small, or unrepresentative samples which can limit the generalisability of their findings (57, 58, 60, 62–64), one had data collection issues such as gaps in pharmacy data requiring ongoing manual data review (57) or non-verbatim transcription of native language discussions (61), one study described a mobile health record intervention that is currently in the research protocol development stage (60), and four studies were unclear about ethical considerations with inadequate consent and confidentiality information (56, 57, 59, 65). Despite the methodological limitations of the included studies, conducting this review remains essential for providing a comprehensive understanding of the topic.

This review has limitations in relation to its scope and methodology. For instance, the search was restricted to the English language for practical reasons, which may have resulted in the exclusion of pertinent studies published in other languages. Furthermore, as research on mobile health record app is rapidly evolving, it is possible that some recent publications were not captured in our review. Lastly, this study emphasised the most significant findings from the literature about the functionalities and features of mobile health record apps that support PESE for promoting trauma-informed and integrated care. Nevertheless, it is impossible to address all of the aspects we found in relation to the implementation of mobile health record apps in healthcare settings to promote trauma-informed and integrated care in a limited space. Acknowledging these limitations allows for a more nuanced interpretation of the findings while still contributing valuable insights to the existing body of knowledge (69).

The strength of this study lies in its ability to examine the functionalities and features of mobile health record apps that support PESE in their implementation context and resultant outcomes. The majority of research on mobile health record apps does not specifically look at their use by PESE or social workers working with PESE. Furthermore, research into mobile health record apps has focused on narrow outcomes like medication adherence for persons experiencing chronic conditions, rather than more complex outcomes, such as integration of care or implementation of the principles of trauma-informed care. This study contributes to the existing body of literature by illustrating the characteristics of the limited number of studies that have examined the implementation of mobile health record apps in their respective healthcare settings for PESE.

## Conclusion

The primary objective of this review was to evaluate the functionalities and features of mobile health record apps that support PESE in relation to their reported outcomes and the delivery of trauma-informed and/or integrated healthcare. Secondary objectives included: (i) identifying the primary users of these apps and describing their recruitment methods, and (ii) assessing key app features, with particular focus on multiplatform accessibility and interoperability within healthcare systems. Although there is growing evidence on this topic, more research is needed, especially concerning persons experiencing homelessness and indigenous populations. Four key themes emerged from the literature: (i) support for integrated and connected care; (ii) enhancement of user engagement and care coordination; (iii) improvement of data accuracy and access to care; and (iv) provision of ongoing monitoring and feedback. Importantly, none of the studies reviewed were implemented within their respective health settings using a trauma-informed approach to promote integrated care. Additionally, few app functionalities aligned with the six principles of the trauma-informed computing framework. It is essential to recognise that these mobile health record apps do not function as isolated interventions; rather, their effectiveness depends on the specific healthcare contexts and conditions in which they are used. PESE were typically the primary users of these apps, while healthcare professionals – including social workers and community health workers – acted as secondary users, or vice versa. Most of the studies used existing channels to recruit the participants and a few studies were unclear about it. Only a few mobile health record apps demonstrated integration with existing health information technologies, and most studies did not clearly specify whether the apps were compatible with iOS or Android platforms. The next step in this research is to use these findings to inform the design of a survey instrument and interview questions for key stakeholders. This will help formulate a comprehensive set of end-user requirements for developing a mobile health record app to be implemented in the hospital from a trauma-informed manner to promote integrated care. This process of firstly developing an understanding of functionalities and features of these apps in their respective implementation contexts supports the creation of a rich bank of questions that will add to the development of a set of end-user requirements. That will help identify practical and effective strategies for PESE in accessing healthcare in the future.

## Data Availability

The original contributions presented in the study are included in the article/Supplementary Material, further inquiries can be directed to the corresponding author.
