# Supplementary material for: What are the functionalities and features of mobile health record apps supporting persons experiencing social exclusion? A systematic literature review
Source: Front Digit Health. 2025 Sep 15;7:1629289. doi: 10.3389/fdgth.2025.1629289 (PMC12477232; doi:10.3389/fdgth.2025.1629289)
Supplement: Supplementary file 1 [file Datasheet1.pdf]

Supplementary Material

1 Supplementary File 1: Search strategy for which mobile record apps support socially excluded individuals

EMBASE [225]

'mobile health application'/exp OR 'mobile application'/exp

((mobile NEAR/2 health) OR mHealth OR m-Health):ti,ab,kw

((smartphone* OR 'cell phone*') NEAR/3 app*):ti,ab,kw

#1 OR #2 OR #3

'electronic health record'/exp OR 'medical record'/exp OR 'patient information'/exp

((medical OR health OR virtual OR electronic OR digital OR mobile) NEAR/3 ('health record*' OR 'medical record*' OR 'patient* record*' OR 'patient* history*' OR 'patient* information')):ti,ab,kw

('My health record' OR myhealthrecord OR myhr OR 'personally controlled electronic health record*' OR e-record OR EMR OR EMRs OR EHR OR 'EHRs'):ti,ab,kw

#5 OR #6 OR #7

'social exclusion'/exp/mj OR 'social discrimination'/exp

(Social* NEAR/2 (justice OR exclusion OR excluded OR marginalised OR marginalized OR disadvantage* OR inclusi*)):ti,ab,kw

('diversity equity and inclusion' OR underserved OR 'multiple disadvantages' OR 'hidden disabilit*'):ti,ab,kw

'health disparity'/exp OR 'health equity'/exp

((health OR socioeconomic) NEAR/2 (disparit* OR equit* OR inequit* OR inequalit*)):ti,ab,kw

'homelessness'/exp OR 'homeless person'/exp

homeless*:ti,ab,kw

(Rough NEAR/3 sleep*):ti,ab,kw

((Insecur* OR inadequate OR precarious OR instability) NEAR/3 (housing OR accommodation)):ti,ab,kw

'forced migrant'/exp

((displaced or undocumented) NEAR/3 (person* OR people OR immigrant* OR population*)):ti,ab,kw

('forced migrant*' OR ‘migrant worker*’ OR 'forced migration*' OR refugee* OR 'asylum seeker*' OR 'unaccompanied minor*'):ti,ab,kw

'addiction'/exp OR 'substance abuse'/exp OR 'substance use'/exp OR 'alcoholism'/exp

(addict* OR alcoholi*):ti,ab,kw

((substance OR drug) NEXT/1 (abuse* OR use*)):ti,ab,kw

#9 OR #10 OR #11 OR #12 OR #13 OR #14 OR #15 OR #16 OR #17 OR #18 OR #19 OR #20 OR #21 OR #22 OR #23

#4 AND #8 AND #24

Medline [144]

Mobile Applications/ or exp Cell Phones/

((mobile adj2 health) OR mHealth OR m-Health).tw.

((Smartphone* OR Cell Phone*) adj3 app*).tw.

1 OR 2 OR 3

exp Electronic Health Records/ OR exp Medical Records/

((medical OR health OR virtual OR electronic OR digital OR mobile) adj3 (health record* OR medical record* OR patient* record* OR patient* histor* OR patient* information)).tw.

(My health record OR myhealthrecord OR myhr OR personally controlled electronic health record* OR e-record OR EMR OR EMRs OR EHR OR EHRs).tw.

5 OR 6 OR 7

exp Social Isolation/ OR exp Socioeconomic Factors/

(Social* adj2 (justice OR exclusion OR excluded OR marginalised OR marginalized OR disadvantage* OR inclusi*)).tw.

("diversity equity and inclusion" OR underserved OR multiple disadvantages OR hidden disabilit*).tw.

Healthcare Disparities/ OR exp Health Inequities/

((health OR socioeconomic) adj2 (disparit* OR equit* OR inequit* OR inequalit*)).tw.

exp Homeless Persons/

homeless*.tw.

(Rough adj3 sleep*).tw.

((Insecur* OR inadequate OR precarious OR instability) adj3 (housing OR accommodation)).tw.

Refugees/ or "Transients and Migrants"/

((displaced or undocumented) adj3 (person* OR people OR immigrant* OR population*)).tw.

(forced migrant* OR migrant worker* OR forced migration* OR refugee* OR asylum seeker* OR refugee* OR unaccompanied minor* OR illegal alien* OR illegal immigrant* OR unauthorized immigrant* OR undocumented alien* OR undocumented immigrant*).tw.

exp Substance-Related Disorders/

(addict* OR alcoholi*).tw.

((substance OR drug) adj1 (abuse* OR use* OR dependence)).tw.

9 OR 10 OR 11 OR 12 OR 13 OR 14 OR 15 OR 16 OR 17 OR 18 OR 19 OR 20 OR 21 OR 22 OR 23

4 AND 8 AND 24

CINAHL [73]

(MH "Mobile Applications") OR (MH "Smartphone") OR (MH "Cellular Phone")

TI ("mobile health" OR mHealth OR m-Health) OR AB ("mobile health" OR mHealth OR m-Health)

TI (Smartphone* N3 app*) OR AB (Smartphone* N3 app*)

S1 OR S2 OR S3

(MH "Electronic Health Records") OR (MH "Medical Records, Personal") OR (MH "Patient Discharge Summaries")

TI ((Medical OR health OR virtual OR electronic) N3 record*) OR AB ((Medical OR health OR virtual OR electronic) N3 record*)

TI ("medical history" OR e-record OR EMR OR EMRs OR EHR OR EHRs) OR AB ("medical history" OR e-record OR EMR OR EMRs OR EHR OR EHRs)

S5 OR S6 OR S7

(MH "Social Deprivation") OR (MH "Discrimination+") OR (MH "Healthcare Disparities") OR (MH "Health Services Accessibility") OR (MH "Diversity, Equity, Inclusion") OR (MH "Homeless Persons") OR (MH "Homelessness") OR (MH "Refugees") OR (MH "Emigration and Immigration") OR (MH "Immigrants+") OR (MH "Substance Use Disorders") OR (MH "Social Problems+") OR (MH "Behavior, Addictive") OR (MH "Substance Dependence") OR (MH "Alcoholism") OR (MH "Alcohol Abuse")

TI (Social* N2 (justice OR exclusion OR excluded OR marginalised OR marginalized OR disadvantage* OR inclusi* OR discriminat*)) OR AB (Social* N2 (justice OR exclusion OR excluded OR marginalised OR marginalized OR disadvantage* OR inclusi* OR discriminat*))

TI ("diversity equity and inclusion" OR underserved OR "multiple disadvantages" OR "hidden disabilit*") OR AB ("diversity equity and inclusion" OR underserved OR "multiple disadvantages" OR "hidden disabilit*")

TI (Health N1 (disparit* OR equit* OR inequit* OR inequalit*)) OR AB (Health N1 (disparit* OR equit* OR inequit* OR inequalit*))

TI (homeless*) OR AB (homeless*)

TI (Rough N3 sleep*) OR AB (Rough N3 sleep*)

TI ((Insecur* OR inadequate OR precarious OR instability) N3 (housing OR accommodation)) OR AB ((Insecur* OR inadequate OR precarious OR instability) N3 (housing OR accommodation))

TI ((displaced or undocumented) N3 (person* OR people OR immigrant* OR population*)) OR AB ((displaced or undocumented) N3 (person* OR people OR immigrant* OR population*))

TI ("forced migrant*" OR "migrant worker*" OR "forced migration*" OR refugee* OR "asylum seeker*" OR "unaccompanied minor*" OR addict* OR alcoholi*) OR AB ("forced migrant*" OR "migrant worker*" OR "forced migration*" OR refugee* OR "asylum seeker*" OR "unaccompanied minor*" OR addict* OR alcoholi*)

TI ((substance OR drug) N1 (abuse* OR use*)) OR AB ((substance OR drug) N1 (abuse* OR use*))

S9 OR S10 OR S11 OR S12 OR S13 OR S14 OR S15 OR S16 OR S17 OR S18

S4 AND S8 AND S19

PsycINFO [88]

DE "Mobile Applications" OR DE "Mobile Health Applications" OR DE "Mobile Phones" OR DE "Smartphone Use" OR DE "Smartphones"

TI ("mobile health" OR mHealth OR m-Health) OR AB ("mobile health" OR mHealth OR m-Health)

TI (Smartphone* N3 app*) OR AB (Smartphone* N3 app*)

S1 OR S2 OR S3

DE "Electronic Health Records" OR DE "Medical Records" OR DE "Patient History" OR DE "Electronic Health Services"

TI ((Medical OR health OR virtual OR electronic) N3 record*) OR AB ((Medical OR health OR virtual OR electronic) N3 record*)

TI ("medical history" OR e-record OR EMR OR EMRs OR EHR OR EHRs) OR AB ("medical history" OR e-record OR EMR OR EMRs OR EHR OR EHRs)

S5 OR S6 OR S7

DE "Social Justice" OR DE "Social Equality" OR DE "Social Deprivation" OR DE "Social Disadvantage" OR DE "Social Discrimination" OR DE "Homeless" OR DE "Social Issues" OR DE "Disadvantaged" OR DE "Poverty" OR DE "Transitional Housing" OR DE "Drug Abuse" OR DE "Substance Use Disorder" OR DE "Addiction" OR DE "Drug Addiction" OR DE "Drug Usage Screening" OR DE "Equity" OR DE "Equity, Diversity, and Inclusion" OR DE "Health Disparities" OR DE "Racial Disparities" OR DE "Social Equity" OR DE "Socioeconomic Disparities" OR DE "Diversity"

TI (Social* N2 (justice OR exclusion OR excluded OR marginalised OR marginalized OR disadvantage* OR inclusi* OR discriminat*)) OR AB (Social* N2 (justice OR exclusion OR excluded OR marginalised OR marginalized OR disadvantage* OR inclusi* OR discriminat*))

TI ("diversity equity and inclusion" OR underserved OR "multiple disadvantages" OR "hidden disabilit*") OR AB ("diversity equity and inclusion" OR underserved OR "multiple disadvantages" OR "hidden disabilit*")

TI (Health N1 (disparit* OR equit* OR inequit* OR inequalit*)) OR AB (Health N1 (disparit* OR equit* OR inequit* OR inequalit*))

TI (homeless*) OR AB (homeless*)

TI (Rough N3 sleep*) OR AB (Rough N3 sleep*)

TI ((Insecur* OR inadequate OR precarious OR instability) N3 (housing OR accommodation)) OR AB ((Insecur* OR inadequate OR precarious OR instability) N3 (housing OR accommodation))

TI ((displaced or undocumented) N3 (person* OR people OR immigrant* OR population*)) OR AB ((displaced or undocumented) N3 (person* OR people OR immigrant* OR population*))

TI ("forced migrant*" OR "migrant worker*" OR "forced migration*" OR refugee* OR "asylum seeker*" OR "unaccompanied minor*" OR addict* OR alcoholi*) OR AB ("forced migrant*" OR "migrant worker*" OR "forced migration*" OR refugee* OR "asylum seeker*" OR "unaccompanied minor*" OR addict* OR alcoholi*)

TI ((substance OR drug) N1 (abuse* OR use*)) OR AB ((substance OR drug) N1 (abuse* OR use*))

S9 OR S10 OR S11 OR S12 OR S13 OR S14 OR S15 OR S16 OR S17 OR S18

S4 AND S8 AND S19

Web of Science Core Collections [64]

("mobile health" OR mHealth OR m-Health) OR (Smartphone* NEAR/3 app*)

((Medical OR health OR virtual OR electronic) NEAR/3 record*) OR ("medical history" OR e-record OR EMR OR EMRs OR EHR OR EHRs)

(Social* NEAR/2 (justice OR exclusion OR excluded OR marginalised OR marginalized OR disadvantage* OR inclusi* OR discriminat*)) OR ("diversity equity and inclusion" OR underserved OR "multiple disadvantages" OR "hidden disabilit*") OR (Health NEAR/1 (disparit* OR equit* OR inequit* OR inequalit*)) OR homeless* OR (Rough NEAR/3 sleep*) OR ((Insecur* OR inadequate OR precarious OR instability) NEAR/3 (housing OR accommodation)) OR ((displaced or undocumented) NEAR/3 (person* OR people OR immigrant* OR population*)) OR "forced migrant*" OR "migrant worker*" OR "forced migration*" OR refugee* OR "asylum seeker*" OR "unaccompanied minor*" OR addict* OR alcoholi* OR ((substance OR drug) NEAR/1 (abuse* OR use*))

#1 AND #2 AND #3

SCOPUS [30]

TITLE-ABS-KEY (("mobile health" OR mHealth OR m-Health) OR (Smartphone* W3 app*))

TITLE-ABS-KEY (((Medical OR health OR virtual OR electronic) W3 record*) OR ("medical history" OR e-record OR EMR OR EMRs OR EHR OR EHRs))

TITLE-ABS-KEY ((Social* W/2 (justice OR exclusion OR excluded OR marginalised OR marginalized OR disadvantage* OR inclusi* OR discriminat*)) OR "diversity equity and inclusion" OR underserved OR "multiple disadvantages" OR "hidden disabilit*" OR (Health W/1 (disparit* OR equit* OR inequit* OR inequalit*)) OR homeless* OR (Rough W/3 sleep*) OR ((Insecur* OR inadequate OR precarious OR instability) W/3 (housing OR accommodation)) OR ((displaced or undocumented) W/3 (person* OR people OR immigrant* OR population*)) OR "forced migrant*" OR "migrant worker*" OR "forced migration*" OR refugee* OR "asylum seeker*" OR "unaccompanied minor*" OR addict* OR alcoholi* OR ((substance OR drug) W/1 (abuse* OR use*)))

#1 AND #2 AND #3

Cochrane [877]

[mh "Mobile Applications"] or [mh "Cell Phones"]

((mobile NEAR/2 health) OR mHealth OR m-Health):ti,ab,kw

((smartphone OR smartphones OR "cell phone") NEAR/3 app*):ti,ab,kw

#1 OR #2 OR #3

[mh "Electronic Health Records"] OR [mh "Medical Records"]

((medical OR health OR virtual OR electronic OR digital OR mobile) NEAR/3 ('health record*' OR 'medical record' OR 'patient record' OR 'patient history' OR 'patient information')):ti,ab,kw

("My health record" OR myhealthrecord OR myhr OR "personally controlled electronic health record" OR e-record OR EMR OR EMRs OR EHR OR "EHRs"):ti,ab,kw

#5 OR #6 OR #7

[mh "Social Isolation"] OR [mh "Socioeconomic Factors"]

(Social* NEAR/2 (justice OR exclusion OR excluded OR marginalised OR marginalized OR disadvantage* OR inclusi*)):ti,ab,kw

("diversity equity and inclusion" OR underserved OR "multiple disadvantages" OR "hidden disability"):ti,ab,kw

[mh "Healthcare Disparities"] OR [mh "Health Inequities"]

((health OR socioeconomic) NEAR/2 (disparit* OR equit* OR inequit* OR inequalit*)):ti,ab,kw

[mh "Homeless Persons"]

homeless*:ti,ab,kw

(Rough NEAR/3 sleep*):ti,ab,kw

((Insecur* OR inadequate OR precarious OR instability) NEAR/3 (housing OR accommodation)):ti,ab,kw

[mh "Refugees"] OR [mh "Transients and Migrants"]

((displaced or undocumented) NEAR/3 (person* OR people OR immigrant* OR population*)):ti,ab,kw

("forced migrant" OR "migrant worker" OR "forced migration" OR refugee* OR "asylum seekers" OR "unaccompanied minors"):ti,ab,kw

[mh "Substance-Related Disorders"]

(addict* OR alcoholi*):ti,ab,kw

((substance OR drug) NEXT/1 (abuse* OR use*)):ti,ab,kw

#9 OR #10 OR #11 OR #12 OR #13 OR #14 OR #15 OR #16 OR #17 OR #18 OR #19 OR #20 OR #21 OR #22 OR #23

#4 AND #8 AND #24

ProQuest Dissertations & Theses A&I [51]

("mobile health" OR mHealth OR m-Health) OR (Smartphone* N/3 app*)

((Medical OR health OR virtual OR electronic) N/3 record*) OR ("medical history" OR e-record OR EMR OR EMRs OR EHR OR EHRs)

(Social* N/2 (justice OR exclusion OR excluded OR marginalised OR marginalized OR disadvantage* OR inclusi* OR discriminat*)) OR ("diversity equity and inclusion" OR underserved OR "multiple disadvantages" OR "hidden disabilit*") OR (Health N/1 (disparit* OR equit* OR inequit* OR inequalit*)) OR homeless* OR (Rough N/3 sleep*) OR ((Insecur* OR inadequate OR precarious OR instability) N/3 (housing OR accommodation)) OR ((displaced or undocumented) N/3 (person* OR people OR immigrant* OR population*)) OR "forced migrant*" OR "migrant worker*" OR "forced migration*" OR refugee* OR "asylum seeker*" OR "unaccompanied minor*" OR addict* OR alcoholi* OR ((substance OR drug) N/1 (abuse* OR use*))

#1AND #2 AND #

2 Supplementary File 2: QATSDD scores

		**Score (0-3)**										
**Criteria**		Ashworth et al. (2022)	Borsari et al. (2018)	Claborn et al. (2017)	Edgar et al. (2022)	Lyles et al. (2023)	Miah et al. (2017)	Moore et al. (2014)	Quanbeck et al. (2018)	Xu et al. (2021)	Zaidi et al. (2020)	Zhou et al. (2017)
1	Explicit theoretical framework	3	3	3	0	0	3	0	3	3	0	0
2	Statement of aims/objectives in main body of report	3	3	3	3	3	3	3	3	3	3	3
3	Clear description of research setting	3	2	2	2	2	2	2	3	2	2	2
4	Evidence of sample size considered in terms of analysis	2	1	1	2	2	3	2	2	2	3	1
5	Representative sample of target group of a reasonable size	2	1	2	2	2	2	3	2	1	1	1
6	Description of procedure for data collection	3	2	2	2	2	3	2	3	2	2	2
7	Rationale for choice of data collection tool(s)	2	2	1	2	2	2	2	2	2	2	2
8	Detailed recruitment data	1	2	2	2	1	1	2	2	2	2	1

9	Statistical assessment of reliability and validity of measurement tool(s) (Quantitative only)	N/A	1	1	N/A	1	3	N/A	2	N/A	2	3
10	Fit between stated research question and method of data collection (Quantitative only)	N/A	2	2	N/A	1	2	N/A	3	N/A	2	2
11	Fit between stated research question and format and content of data collection tool e.g. interview schedule (Qualitative only)	3	N/A	3	2	2	2	N/A	N/A	3	N/A	N/A
12	Fit between research question and method of analysis (Quantitative only)	N/A	2	2	N/A	2	3	2	N/A	N/A	2	3
13	Good justification for analytic method selected	2	2	2	2	1	3	2	2	2	2	3
14	Assessment of reliability of analytic process (Qualitative only)	1	N/A	2	N/A	2	1	N/A	1	2	N/A	N/A
15	Evidence of user involvement in design	3	1	3	3	1	3	2	2	2	1	3
16	Strengths and limitations critically discussed	2	2	2	2	2	2	2	2	2	2	2
	Total	30	26	33	24	25	38	24	32	24	24	28

3 Supplementary File 3: Results for Mixed Methods Appraisal Tool (MMAT)

**Category of study designs**	**Methodological quality criteria**	Ashworth et al. (2022)	Borsari et al. (2018)	Claborn et al. (2017)	Edgar et al. (2022)	Lyles et al. (2023)	Miah et al. (2017)	Moore et al. (2014)	Quanbeck et al. (2018)	Xu et al. (2021)	Zaidi et al. (2020)	Zhou et al. (2017)
Screening questions<br>(for all types)	S1. Are there clear research questions?	Yes	Yes	Yes	Yes	Yes	Yes	Yes	Yes	Yes	Yes	Yes
	S2. Do the collected data allow to address the research questions?	Yes	Yes	Can't tell	Yes	Yes	Yes	Yes	Yes	Yes	Yes	Yes
	Further appraisal may not be feasible or appropriate when the answer is 'No' or 'Can't tell' to one or both screening questions.											
1. Qualitative	1.1. Is the qualitative approach appropriate to answer the research question?	Yes				Yes					Yes	Yes
	1.2. Are the qualitative data collection methods adequate to address the research question?	Yes				Yes					Yes	Yes
	1.3. Are the findings adequately derived from the data?	Yes				Yes					Yes	Yes
	1.4. Is the interpretation of results sufficiently substantiated by data?	Yes				Yes					Yes	Yes
	1.5. Is there coherence between qualitative data sources, collection, analysis and interpretation?	Yes				Yes					Yes	Yes
2. Quantitative randomized controlled trials	2.1. Is randomization appropriately performed?									Yes		
	2.2. Are the groups comparable at baseline?									Yes		
	2.3. Are there complete outcome data?									No		
	2.4. Are outcome assessors blinded to the intervention provided?									No		

	2.5 Did the participants adhere to the assigned intervention?									Yes		
3. Quantitative non-randomised	3.1. Are the participants representative of the target population?								Can't tell			
	3.2. Are measurements appropriate regarding both the outcome and intervention (or exposure)?								Yes			
	3.3. Are there complete outcome data?								Can't tell			
	3.4. Are the confounders accounted for in the design and analysis?								No			
	3.5. During the study period, is the intervention administered (or exposure occurred) as intended?								Yes			
4. Quantitative descriptive	4.1. Is the sampling strategy relevant to address the research question?		Yes			Yes	Yes					
	4.2. Is the sample representative of the target population?		Can't tell			Can't tell	Can't tell					
	4.3. Are the measurements appropriate?		Yes			Yes	Yes					
	4.4. Is the risk of nonresponse bias low?		Can't tell			Can't tell	Can't tell					
	4.5. Is the statistical analysis appropriate to answer the research question?		Yes			Yes	Yes					
5. Mixed methods	5.1. Is there an adequate rationale for using a mixed methods design to address the research question?	Yes				Can't tell	No	Yes				
	5.2. Are the different components of the study effectively integrated to answer the research question?	Yes				Can't tell	No	Yes				

	5.3. Are the outputs of the integration of qualitative and quantitative components adequately interpreted?	Can’t tell				Can’t tell	No	Yes				
	5.4. Are divergences and inconsistencies between quantitative and qualitative results adequately addressed?	Can’t tell				Can’t tell	No					
	5.5. Do the different components of the study adhere to the quality criteria of each tradition of the methods involved?	Yes				Can’t tell	Yes	Yes				
